# Cognitive decline, Aβ pathology, and blood–brain barrier function in aged 5xFAD mice

**DOI:** 10.1186/s12987-024-00531-x

**Published:** 2024-03-27

**Authors:** Geetika Nehra, Sasivimon Promsan, Ruedeemars Yubolphan, Wijitra Chumboatong, Pornpun Vivithanaporn, Bryan J. Maloney, Anusorn Lungkaphin, Bjoern Bauer, Anika M. S. Hartz

**Affiliations:** 1 Sanders-Brown Center On Aging, University of Kentucky, 760 Press Ave, 124 HKRB, Lexington, KY 40536-0679 USA; 2 Department of Physiology, Faculty of Medicine, Chiang Mai University, Chiang Mai, Thailand; 3 Department of Pharmacology, Faculty of Medicine, Chiang Mai University, Chiang Mai, Thailand; 4 Faculty of Medicine Ramathibodi Hospital, Chakri Naruebodindra Medical Institute, Mahidol University, Nakhon Pathom, Thailand; 5 Department of Pharmaceutical Sciences, College of Pharmacy, University of Kentucky, Lexington, USA; 6 Department of Pharmacology and Nutritional Sciences, College of Medicine, University of Kentucky, Lexington, USA

**Keywords:** 5xFAD, Blood–Brain Barrier, Aβ, *Pde6b*^*rd1*^

## Abstract

**Background:**

Patients with Alzheimer's disease (AD) develop blood–brain barrier dysfunction to varying degrees. How aging impacts Aβ pathology, blood–brain barrier function, and cognitive decline in AD remains largely unknown. In this study, we used 5xFAD mice to investigate changes in Aβ levels, barrier function, and cognitive decline over time.

**Methods:**

5xFAD and wild-type (WT) mice were aged between 9.5 and 15.5 months and tested for spatial learning and reference memory with the Morris Water Maze (MWM). After behavior testing, mice were implanted with acute cranial windows and intravenously injected with fluorescent-labeled dextrans to assess their in vivo distribution in the brain by two-photon microscopy. Images were processed and segmented to obtain intravascular intensity, extravascular intensity, and vessel diameters as a measure of barrier integrity. Mice were sacrificed after in vivo imaging to isolate brain and plasma for measuring Aβ levels. The effect of age and genotype were evaluated for each assay using generalized or cumulative-linked logistic mixed-level modeling and model selection by Akaike Information Criterion (AICc). Pairwise comparisons were used to identify outcome differences between the two groups.

**Results:**

5xFAD mice displayed spatial memory deficits compared to age-matched WT mice in the MWM assay, which worsened with age. Memory impairment was evident in 5xFAD mice by 2–threefold higher escape latencies, twofold greater cumulative distances until they reach the platform, and twice as frequent use of repetitive search strategies in the pool when compared with age-matched WT mice. Presence of the *rd1* allele worsened MWM performance in 5xFAD mice at all ages but did not alter the rate of learning or probe trial outcomes. 9.5-month-old 15.5-month-old 5xFAD mice had twofold higher brain Aβ_40_ and Aβ_42_ levels (*p* < 0.001) and 2.5-fold higher (*p* = 0.007) plasma Aβ_40_ levels compared to 9.5-month-old 5xFAD mice. Image analysis showed that vessel diameters and intra- and extravascular dextran intensities were not significantly different in 9.5- and 15.5-month-old 5xFAD mice compared to age-matched WT mice.

**Conclusion:**

5xFAD mice continue to develop spatial memory deficits and increased Aβ brain levels while aging. Given in vivo MP imaging limitations, further investigation with smaller molecular weight markers combined with advanced imaging techniques would be needed to reliably assess subtle differences in barrier integrity in aged mice.

## Background

Alzheimer’s disease (AD) is the most common form of dementia and accounts for more than 6.5 million cases. It incurs direct costs exceeding $300 billion in healthcare and long-term care in the United States [1]. These numbers will likely double by 2050 and highlight a critical unmet need for disease-modifying therapies for AD patients [2]. To develop an effective disease-modifying therapy for AD patients, investigators have examined how underlying factors such as amyloid deposition, tauopathy, and neurodegeneration contribute to cognitive decline in AD [3, 4]. Recently, blood–brain barrier dysfunction has also been recognized as an underlying factor contributing to patients’ cognitive decline [5–7]. Microvascular dysfunction is an umbrella term that includes changes such as cerebral amyloid angiopathy (CAA), pericyte degeneration, capillary stalling, reduced cerebral blood flow, string vessel formation, reduced vessel density or capillary leakage detected by dynamic contrast-enhanced-magnetic resonance imaging (MRI), or positron emission tomography (PET) scans [8–11]. Increasing evidence indicates that Aβ contributes to microvascular dysfunction; however, the underlying mechanism for this observation is unknown [12–15]. In the present study, we used the 5xFAD mouse model to investigate the effect of aging and Aβ levels on microvascular dysfunction in AD. 5xFAD mice are widely used in preclinical studies since they overexpress human Aβ proteins and display early and aggressive Aβ pathology prior to neuronal loss, gliosis, Aβ plaques, synaptic loss, barrier dysfunction, and cognitive impairment [16–18]. Previous studies show that 5xFAD mice also develop meningeal cerebral amyloid angiopathy (CAA), reduced cerebral blood flow, and stalled cortical capillaries due to increased adhesion of neutrophils by 6 months of age [19–21]. Despite these known deficits, no conclusive evidence exists for Aβ-driven microvascular dysfunction in aged 5xFAD mice. The effect of aging on Aβ levels, cognition, and blood–brain barrier function is also not well-characterized in this mouse model. In this study, we aged 5xFAD mice to 9.5 and 15.5 months so that these mice display Aβ accumulation. We further tested our hypothesis that Aβ-driven microvascular dysfunction is age-dependent.

## Materials & methods

### Animals

Animal experiments were approved by the Institutional Animal Care and Use Committee at the University of Kentucky (#2014–1233 and #2020–3455; PI: Hartz) and were in accordance with AAALAC regulations, the Guide of the Care and Use of Laboratory Animals, and the US Department of Agriculture Animal Welfare Act. Male transgenic 5xFAD mice (Tg6799; B6SJL-Tg (APPSwFlLonPSEN1*M146L*L286V)6799Vas/Mmjax; MMRRC Strain: 034840-JAX) and corresponding non-carrier littermates (WT mice) were obtained from Mutant Mouse Resource & Research Center (MMRRC) supported by the NIH at Jackson Laboratory (Bar Harbor, ME, USA). Animals were received at 4–8 weeks of age and group-housed in an AAALAC-accredited temperature-and-humidity-controlled vivarium with standard chow feed (Teklad 2918; Inotiv, West Lafayette, IN, USA) and water ad libitum under a 12 h light/dark cycle (temperature: 25 ± 1 °C; humidity: 50 ± 5%). Mice were genotyped for APPswe transgene and native *Pde6b*^*rd1*^ allele by polymerase chain reaction analysis (Transnetyx, Cordova, TN, USA) of ear and brain tissue samples and were aged 9–10 months and 15–16 months before experimentation. *Pde6b*^*rd1*^ status was assessed because this allele as a homozygote causes severe retinal deterioration and heterozygotes have visual deficiency [22]. 5xFAD and WT mice with heterozygous *Pde6b*^*rd1*^ allele are referred to as 5xFAD^*rd1/wt*^ and WT^*rd1/wt*^ mice in this study. 5xFAD and WT mice without *Pde6b*^*rd1*^ allele are referred to as 5xFAD^*wt1/wt*^ and WT^*wt1/wt*^ mice in this study. 133 mice were allocated to this study (*n* = 50 for 15.5-month-old 5xFAD mice, *n* = 33 for 15.5-month-old WT mice, n = 25 for 9.5-month-old 5xFAD mice, *n* = 25 for 9.5-month-old WT mice). Of these, 71 mice survived till endpoint (*n* = 18 for 15.5-month-old 5xFAD mice, *n* = 13 for 15.5-month-old WT mice, *n* = 21 for 9.5-month-old 5xFAD mice, *n* = 19 for 9.5-month-old WT mice). Assessment of *rd1* allele revealed uneven distribution across ages and genotypes (Table 1; χ^2^ = 32.875;* p* = 0.492).

**Table 1:** Distribution of Rd1 mutation in mice used for MWM test

Age	WT (RD-)	WT (RD +)	5xFAD (RD-)	5xFAD (RD +)
9.5 months	5	5	4	3
15.5 months	2	8	8	6

Mice were genotyped for the presence/absence of the *rd1* allele. Results are tabulated by age and transgene status

### Morris water maze test

**Fig. 1:**
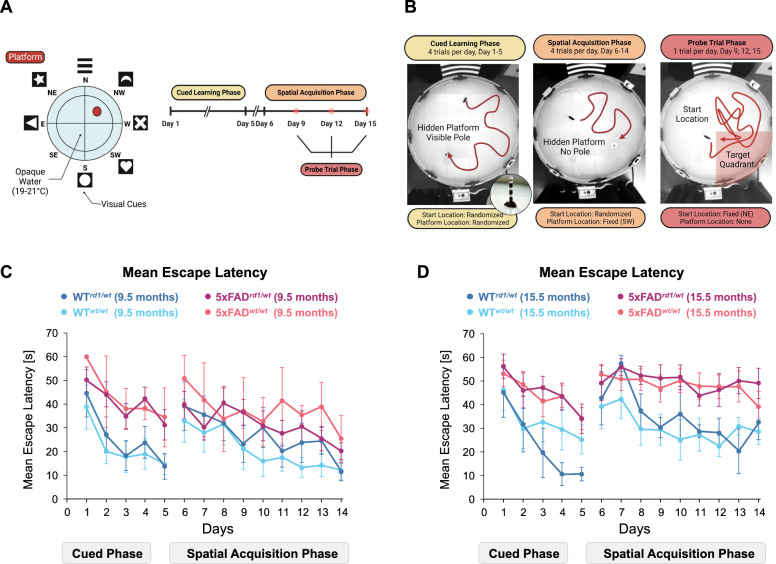
Learning and Spatial Memory Impairments in Aged 5xFAD and WT mice. **A** Schematic diagram for MWM cued learning phase, spatial acquisition phase, and MWM probe trials. **B** Pictures of mice searching the platform during the cued learning phase, spatial acquisition phase, and probe trial. Insert shows a mouse on the platform marked with a pole at the end of a trial. **C** Censored mean escape latency (s) for 9.5-month-old WT^*rd1/wt*^ mice (dark blue circles), 9.5-month-old WT^*wt/wt*^ mice (light blue circles), 9.5-month-old 5xFAD^*rd1/wt*^ mice (magenta circles), and 9.5-month-old ^*wt//wt*^ mice (red circles). D) Censored mean escape latency (s) for 15.5-month-old WT^*rd1/wt*^ mice (dark blue circles), 15.5-month-old WT^*wt/wt*^ mice (light blue circles), 15.5-month-old 5xFAD^*rd1/wt*^ mice (magenta circles), and 15.5-month-old ^*wt//wt*^ mice (red circles)

We evaluated 5xFAD and age-matched WT mice for learning and memory deficits using the Morris Water Maze (MWM) test (Fig. 1) as previously described [23]. Prior to behavior testing, mice were handled for 7 days. For testing, mice were introduced to a temperature-controlled, circular pool (diameter: 150 cm; height: 55 cm; temperature: 19–21 °C) surrounded by four tall (40 cm) and four small (20 cm), randomly assigned visual cues. The pool was filled with tap water that was made opaque with non-toxic white paint (Crayola^®^ non-toxic tempera paint; Easton, PA, USA). A circular rescue platform (diameter: 11 cm; 0.5 cm) was submerged 1–1.5 cm below the platform surface. Lighting was adjusted to 50 ± 5 lx. Mice were trained to find the hidden platform over 15 days, 4 trials per day, with a 30 min inter-trial duration. Trials ended if a mouse reached the platform or failed to reach the platform within 60 s. In the latter case, mice were guided to the platform with a ruler. Mice were trained to sit on the platform for 5 s or longer before they were moved out of the pool. Animals that did not reach the platform during the trial were held by the tail on the platform for 20 s. The 15 day test phase was divided into three phases: the cued learning phase (days 1–5), the spatial acquisition phase (days 6–15), and the probe trial phase (days 9, 12, 15). In the cued learning phase, mice were introduced to the pool from randomly assigned starting locations, and a black-and-white striped pole (12 cm height with 1 cm black and white stripes) above the hidden platform served as an additional visual cue for mice to find the platform. In the spatial acquisition phase (days 6–14), the pole was removed, and mice were tested to find the hidden platform at a fixed location (southwest quadrant, SW). In the probe trial phase (days 9, 12, 15), mice were placed into the water in the northeast (NE) quadrant to swim freely for 60 s. Animal performance during each trial was recorded using the automated behavioral video tracking software ANY-maze (Stoelting, Wood Dale, IL, USA).

### Acute cranial window installation

Acute cranial windows were installed in mice based on a modified procedure described by Mostany and Portera-Cailliau [24]. Briefly, mice were anesthetized by isoflurane inhalation (0.7–3%) using the SomnoSuite^®^ anesthesia system (SS409B, Kent Scientific, Torrington, CT, USA). Anesthetic depth was confirmed by unresponsiveness to toe pinch. Anesthetized mice were transferred to a surgical board and secured in the mouth with incisor bars and non-penetrating ear bar adaptors (Kent Scientific, Torrington, CT, USA). Animal body temperature was maintained using a homeothermic blanket and a rectal probe connected to the SomnoSuite^®^ system. The animal’s eyes were covered with an artificial tear ointment (GenTeal^®^, Alcon, Fort Worth, TX, USA). A midline scalp incision was made 3–4 mm caudal to the skull, advancing forward between the eyes. Adherent connective tissue was removed with cotton swabs. Next, a circle (4 mm diameter) was drilled using a hand-held drill connected to a high-speed rotary micromotor control box (Foredom K.1070, Blackstone Industries, Bethel, CT, USA) until the periosteum was visible. At this step, saline was applied to the skull, and the excised skull piece was removed using Dumont #5 forceps (11254–20, Fine Science Tools, Foster City, CA, USA). A coverslip (1, 0.15 mm thickness, 5–8 mm diameter; Harvard Apparatus, Cambridge, MA, USA) was secured to the craniotomized surfaces with cyanoacrylate (Thin Viscosity Cyanoacrylate Adhesive, GlueMasters LLC, Seattle, WA, USA). Cyanoacrylate was then cured with 1–2 drops of 0.9% sterile saline solution (C1880725, Covetrus, Dublin, OH, USA).

### In vivo* two-photon microscopy*

Following acute cranial window installations, anesthetized animals were transferred to the stage of an upright Zeiss LSM 880 confocal microscope (Carl Zeiss Microscopy, White Plains, NY, USA) equipped with a 20x, NA 1.0 water immersion objective, an InSight X3 dual beam extended laser set to 947 nm, another fixed laser set to 1045 nm, and an external binary GaAsP (BiG) NDD detector. A layer of transparent artificial tear gel (GenTeal^®^ Tears Lubricant Eye Gel, Alcon, Fort Worth, TX, USA) was applied on top of the coverslips to provide the necessary refractive index medium for in vivo imaging. Prior to imaging, the animal was intraperitoneally injected with 0.9% sterile saline solution (C1880725, Covetrus, Dublin, OH, USA), followed by a tail vein injection of TexasRed-labeled 3 kDa dextran (TR-3 kDa dextran; Thermo Fisher Scientific, Waltham, MA, USA; 0.1 mg/g body weight) combined with Fluorescein Isothiocyanate-labeled 70 kDa dextran (FITC-70 kDa dextran; Millipore-Sigma, Burlington, MA, USA); 0.4 mg/g body weight). ROIs (0.28 × 0.28 × 0.15 mm) were randomly selected within 10 min of dextran injection and imaged as sequential Z-stacks (30 slices, 3.05 µm step size, 1024 pixels × 1024 pixels; 6 min imaging time) for 70 min. Laser power, digital gain, and digital offset were optimized for the imaging of each animal. Mice were euthanized after in vivo imaging by CO_2_ inhalation followed by decapitation.

### Blood and brain tissue collection

Blood and brain tissue samples were collected using previously described methods [25]. Blood was collected from the right ventricle using a 20G PrecisionGlide^™^ needle (305175, BD Biosciences, Frankin Lakes, NJ, USA) into a 1 mL heparinized PSI microtainer tube (365985; BD Biosciences, Frankin Lakes, NJ, USA). Tubes were gently mixed end-over-end and centrifuged at 10,000 g for 10 min at 4 °C in a Fisherbrand^™^ accuSpin^™^ Micro 17R centrifuge (Fisher Scientific, Waltham, MA, USA). Plasma samples (supernatant) were stored at − 20 °C until further use. Brains were excised, meninges, cerebellum, and olfactory bulbs were removed, and the remaining hemispheres were stored at − 20 °C until further use.

### Aβ extraction

Brain tissue samples from individual 5xFAD mice were weighed; the exact brain weight of each sample was recorded. Then, tissue samples were homogenized in 0.4 mL extraction buffer A (5 M Guanidine-HCl, G3272, Millipore-Sigma, Burlington, MA, USA; 50 mM Tris HCl, T0819, Millipore-Sigma, Burlington, MA, USA) to extract soluble and insoluble Aβ. Homogenates were diluted in extraction buffer B (DBPS, D5652, Millipore-Sigma, Burlington, MA, USA; 5% BSA, A9646, Millipore-Sigma, Burlington, MA, USA; 1 × Protease Inhibitor Cocktail Set 1; Calbiochem, 539131, EMD Millipore, Billerica, MA, USA) and centrifuged at 16,000 g for 20 min at 4 °C. Supernatants containing Aβ were stored at − 20 °C.

### *Aβ*_*40*_* and Aβ*_*42*_* ELISA*

Commercially available human Aβ_40_ (hAβ_40_) ELISA kit (KHB3481; Thermo Fisher Scientific, Waltham, MA, USA) and human Aβ_42_ ELISA kit (KHB3441; Thermo Fisher Scientific) were used to determine Aβ_40_ and Aβ_42_ protein levels in plasma and brain tissue samples of 5xFAD mice as per the manufacturer's instructions. For Aβ_40_ ELISA, plasma samples were used at various dilutions (undiluted, 1:2, 1:4, 1:6, 1:10). Homogenized brain samples were diluted to 1:50,000 and 1:150,000 in assay buffer for Aβ_40_ ELISA and Aβ_42_ ELISA, respectively. Absorbance values were measured at 450 nm using a Synergy H1 microplate reader (Serial 15011310, BioTek^®^ Instruments, Winooski, VT, USA). Standard curves were computed using Gen5 software with a non-linear 4-parameter-logistic regression curve fitting to determine Aβ_40_ and Aβ_42_ concentrations.

### Statistical analysis of escape latency

All analyses herein were performed with the R statistical package [26]. We noted that several trials ended in failure, i.e., a mouse did not locate the platform in 60 s. Thus, the most appropriate analysis would be an accelerated failure time model [27], via the R survival package [28]. Data were clustered by individual animals to account for repeat measures. We compared competing nested models by second-order Akaike information criterion (AICc) [29]. All models included genotype, age, day, and phase. Additional candidate covariates included the presence/absence of the *rd1* allele and potential interactions. After model selection, we performed post hoc fixed-effect comparisons via estimated marginal means and trends [30]. Continuous quantitative covariates were standardized.

### Statistical analysis of search strategy in MWM

We identified search strategy patterns from ANY-maze trial track plots [31]. These strategies can be broadly categorized as spatial, non-spatial, or repetitive looping. Spatial strategies were assigned to trials when mice swam to the platform directly (*spatial direct*), within one loop (*spatial indirect*), or following a focused search in the quadrant containing the platform (*focal correct*). Non-spatial strategies were assigned to trials when mice searched for the platform in the interior portion of the pool (*scanning*), across the entire pool (*random*), or when mice did a focused search in a small portion of the pool that did not contain the platform (*focal incorrect*). Repetitive looping strategies were assigned to trials when mice swam inside the thigmotaxis zone (*peripheral looping*) or in the region interior to the thigmotaxis zone, i.e., 15 cm from the pool's rim (*chaining*). Circling strategies were assigned when mice moved in small tight circles with a directionality. In some trials, mice adopted multiple search strategies over the entire trial duration. In these trials, we selected the strategy used for the longest duration. Search strategies were ranked from least to most structured in the following order: peripheral looping, chaining, circling, random, scanning, focal incorrect, focal correct, spatial indirect, and spatial direct. Data were analyzed by cumulative linked logistic mixed models (clmm) [32–34], with nested random effects to account for repeated measures. Fixed effects, covariates, and interactions were as for latency analysis, and the model was selected by second-order AICc. Continuous quantitative covariates were standardized.

### Moderated mediation analysis of search strategy effect on escape latency

Models derived for latency and strategy analyses were used in moderated mediation analysis [35]. The mediation model included terms for age, day, genotype, phase, and *rd1* status, as well as multiple interactions built from the strategy and latency models. Due to limitations of the R mediation package, a conventional ordinal regression model was substituted for the mixed-level model for strategy. We tested the hypothesis that search strategy mediated the effect of genotype on escape latency, along with moderation effects of age and *rd1* status.

### Statistical analysis of probe trials (MWM Testing)

Probe trial performance was analyzed using (1) percent time in the target quadrant (Q) and (2) mean normalized distance to the platform or proximity (P) [36]. Values were analyzed for (a) the first 15 s and (b) the entire trial duration (60 s). Generalized mixed-level models (glmm) were used to analyze data, with random intercepts by mouse, using the glmmTMB R package [37]. In addition, Q was specifically analyzed by mixed-level ordered beta models via glmmTMB. Ordered beta regression [38] is optimized for analysis of degenerate data with lower and upper bounds, e.g., percentage data that may include 0% and 100% values. This method improves on ordinary least squares, fractional logit, beta regression, and ZOIB (zero/one inflated beta regression), all of which have been used to analyze this sort of data. All candidate models included genotype, age, and r*d1* status, differing by covariates such as test duration (15 vs. 60 s) and day of test, as well as interactions, and were compared by second-order AICc. After model selection, post hoc fixed-effect comparisons were performed by estimating marginal means. Continuous quantitative covariates were standardized. Continuous response variables were standardized to the root mean square.

### Image processing, segmentation and analysis

Acquired Z-stacks were exported as maximum intensity projection (MIP) TIFF files using Fiji/ImageJ (v.1.52; Wayne Rasband, NIH, USA). MIP images were segmented using Zen Intellesis into intravascular and extravascular regions based on a standard segmentation protocol [39]. A training set of 2 images per tracer was used from each animal to create segmentation training models for each animal. Intravascular regions were defined as pixels within and along the vessel lumen for each fluorescent-labeled dextran in the training images from each animal i.e., images at t = 7 min and t = 70 min. Extravascular spaces were defined as pixels classified as background by the training module. All puncta were defined as “extravascular compartment”. Training labels were iteratively refined until the model accurately classified images into intravascular and extravascular compartments. Images were segmented at a minimum threshold intensity of 5000 pixels using ‘Deep Features 50’ and ‘Conditional Random Field’ postprocessing settings, and the following features were exported as a CSV file for each area, sum intensity, and mean intensity. We compared extravascular and intravascular sum intensities for each dextran across groups to assess barrier function. We used mixed-level models with individual animals as random intercepts and laser power and considered genotype, age, time, and dextran size as fixed effects. Final models were selected using second-order AICc. All possible interactions were included in the saturated initial models. Post hoc comparisons of fixed effects were performed by estimating marginal trends.

### Vessel diameter modeling

Vessel diameters from MPI images were manually measured using the line tool in Fiji/ImageJ (v.1.52; Wayne Rasband, NIH, USA). For diameters, we compared mixed-level models with random intercepts by individual animals. We screened out potential random effects for laser power, master gain, digital offset, and digital gain. Age, genotype, and dextran type were included as fixed effects. Final models were selected using second-order AICc. All possible interactions were included in the saturated initial models. Post hoc comparisons of fixed effects were performed by estimating marginal trends.

### Statistical analysis of plasma and brain Aβ levels

Data for Aβ_40_ and Aβ_42_ plasma levels was left-censored (below assay limit) and thus analyzed by Tobit regression. Models with Gaussian and log-normal distributions were compared by second-order AICc. Brain Aβ_40_ and Aβ_42_ levels were analyzed by generalized linear models vs age and genotype.

## Results

### Progression of spatial memory impairment in 5xFAD and WT mice

We first evaluated 5xFAD and age-matched WT mice for learning and memory deficits using the Morris Water Maze (MWM) test (Fig. 1). In this test, we introduced mice into a circular pool, trained them to find a hidden escape platform, and tested their ability to memorize the platform's location. In the cued learning phase (days 1–5), we trained mice to find the hidden escape platform marked with a pole placed on top of the platform (Fig. 1A, B). In the spatial acquisition phase (days 6–14), we trained mice to find a hidden platform with no pole. In both phases, we used escape latency or time taken by the mouse to reach the platform to evaluate spatial learning and cognition [40, 41]. Escape latency in the cued learning phase is based on visual acuity and sensorimotor skills in mice to locate the platform. Escape latency in the spatial acquisition phase shows the animal's ability to use spatial cues to find the submerged platform. Figure 1C and D show a graphical representation of mean escape latencies between 5xFAD mice and age-matched WT mice at 9.5 and 15.5 months of age. Post hoc analysis of estimated marginal means shows that escape latencies increased with age and were higher for 5xFAD mice than age-matched WT mice (*p* < 0.001; Table 2). Escape latencies decreased with each successive day of testing (*p* < 0.001; Table 2). Post hoc analysis also showed that escape latencies in the cued phase significantly decreased with age and each successive day of testing and indicated that older mice learned to reach the platform faster in the cued phase (*p* < 0.001; Table 2). 5xFAD mice took significantly longer to find the platform than age-matched WT mice (*p* < 0.001; Table 2). The presence of the *rd1* allele did not significantly impact escape latencies in MWM test.

**Table 2:** Effect Size Estimates for Escape Latency in MWM test

Effect	Estimate^a^	χ^2^ (df)	*p*	*R*^*2*^
Age	**0.219 ± 0.076**	**87.251 (1)**	** < 0.001**	**0.070**
Genotype (5xFAD)	**0.270 ± 0.081**	**17.141 (1)**	** < 0.001**	**0.127**
*rd1* allele	0.008 ± 0.075	0.119 (1)	0.730	0.009
Test day	− 0.196 ± 0.016	213.881 (1)	< 0.001	0.080
Test phase (Cued)	− 0.070 ± 0.074	1.094 (1)	0.296	0.078
Age × *Rd1* mutation	0.009 ± 0.078	0.146 (1)	0.702	0.004
Genotype × Age	0.034 ± 0.074	2.184 (1)	0.139	0.011
Genotype × *Rd1* mutation	0.028 ± 0.075	1.494 (1)	0.222	0.004
Test Day × Genotype	**0.024 ± 0.010**	**6.912 (1)**	**0.009**	** < 0.001**
Test Phase × Age	− **0.108 ± 0.032**	**22.783 (1)**	** < 0.001**	**0.009**
Test Phase × Genotype	**0.138 ± 0.045**	**12.419 (1)**	** < 0.001**	**0.006**
Test Phase × *Rd1* mutation	**0.043 ± 0.029**	**3.595 (1)**	**0.058**	**0.003**
Test Phase × Test Day	− **0.095 ± 0.016**	**51.594 (1)**	** < 0.001**	**0.017**

Effect size estimates for escape latencies with corresponding *p* values (adjusted by Benjimini-Yakuteli false discovery correction), chi-squared (χ^2^) values, *R*^*2*^ values, and degrees of freedom (df)

^a^Calculated on log link. Data is presented as mean values ± standard error. Relevant effect sizes with *p* < 0.05 are discussed in the results section and shown in bold text

**Fig. 2:**
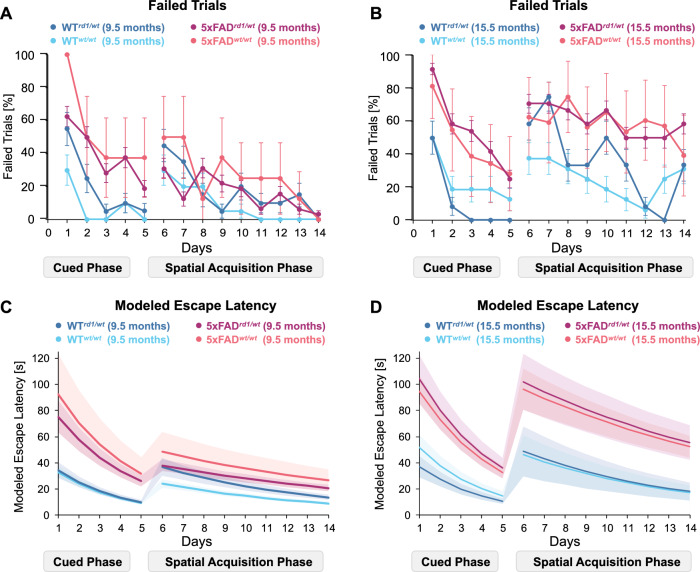
Failed trials in MWM testing and Accelerated Failure Time Modeling. **A** Percentage of failed trials by 9.5-month-old WT^*rd1/wt*^ mice (dark blue circles), 9.5-month-old WT^*wt/wt*^ mice (light blue circles), 9.5-month-old 5xFAD^*rd1/wt*^ mice (magenta circles), and 9.5-month-old^*wt//wt*^ mice (red circles). **B** Percentage of failed trials by 15.5-month-old WT^*rd1/wt*^ mice (dark blue circles), 15.5-month-old WT^*wt/wt*^ mice (light blue circles), 15.5-month-old 5xFAD^*rd1/wt*^ mice (magenta circles), and 15.5-month-old^*wt//wt*^ mice (red circles). **C** Modeled escape latencies for 9.5-month-old WT^*rd1/wt*^ mice (dark blue line), 9.5-month-old WT^*wt/wt*^ mice (light blue line), 9.5-month-old 5xFAD^*rd1/wt*^ mice (magenta line), and 9.5-month-old ^*wt//wt*^ mice (red line). D) Modeled escape latencies for 15.5-month-old WT^*rd1/wt*^ mice (dark blue line), 15.5-month-old WT^*wt/wt*^ mice (light blue line), 15.5-month-old 5xFAD^*rd1/wt*^ mice (magenta line), and 15.5-month-old ^*wt//wt*^ mice (red line). Modeled data is presented as model (solid line) ± 95% prediction intervals (shades). Modeled latencies increased with age and genotype and resulted in an extrapolated estimate for latencies in failed trials

We also noted that mice often failed to reach the platform within the 60 s trial duration, i.e., latencies were right-censored and may not reliably indicate performance in the MWM assay (Fig. 2A, B**)**. In the cued learning phase, 5xFAD mice failed to reach the platform three-times as often as age-matched WT mice (Fig. 2A, B). In the spatial acquisition phase, age-matched 5xFAD and WT mice showed similar trial failure frequencies at 9.5 months of age. In contrast, 15.5-month-old 5xFAD mice failed to reach the platform 1.5-fold times more often compared to age-matched WT mice (Fig. 2A, B). To account for high failure rate of trials, we employed an accelerated time-to-failure model and estimated escape latencies for a hypothetically ‘unending’ trial (Fig. 2C, D). Indeed, modeled latencies were twice as high as the trial duration. Modeled escape latencies were not significantly different across groups except for three instances. First, modeled escape latencies for 9.5-month-old 5xFAD^*rd1/wt*^ mice were 2.4-fold higher (44.18 ± 6.77 s*; p* = 0.002) than those for age-matched WT^*rd1/wt*^ mice (18.18 ± 3.00 s, Table 3, Fig. 2C). Second, modeled escape latencies for 15.5-month-old 5xFAD^*rd1/wt*^ mice were threefold higher (44.18 ± 6.77 s*; p* = 0.002) than those for age-matched WT^*rd1/wt*^ mice (18.18 ± 3.00 s, Table 3, Fig. 2D). Latencies were similar for age-matched 5xFAD^w*t/wt*^ mice and WT^w*t/wt*^ mice, indicating that the *rd1 allele* worsened MWM performance for 5xFAD mice at both ages. Third, modeled escape latencies for 15.5-month-old 5xFAD^*rd1/wt*^ mice (75.04 ± 16.54 s) were threefold higher (*p* = 0.002) than that for younger 5xFAD^*rd1/wt*^ mice (75.04 ± 16.54 s) than at 9.5 months (29.79 ± 3.24 s; Table 3). Latencies did not change with age for 5xFAD^w*t/wt*^ mice, indicating that the *rd1* allele worsened MWM performance in 5xFAD mice with age. In terms of learning rates, 5xFAD mice showed a slower learning rate than WT mice in the cued phase (5xFAD mice: − 0.267 ± 0.032 s; WT mice: − 0.315 ± 0.032 s; *p* = 0.024) and spatial phase (5xFAD mice: − 0.077 ± 0.013 s; WT mice: − 0.125 ± 0.015 s*; p* = 0.024). *Rd1* allele status did not alter slopes in these phases. Taken together, these findings show that the *rd1* allele worsened MWM performance in 5xFAD mice at all ages but did not alter the rate of learning in this cohort.

**Table 3:** Pairwise comparison of modeled escape latencies by age, genotype, *rd1* allele status and learning rates

Modeled escape latencies by genotype					
Phase	Age (m)	*rd1* status	WT Mice	5xFAD Mice	*p*
Cued	9.5	*wt/wt*	17.53 ± 2.39	54.20 ± 19.44	0.061
Cued	**9.5**	***rd1/wt***	**18.18 ± 3.00**	**44.18 ± 6.77**	**0.002**
Cued	15.5	*wt/wt*	27.58 ± 5.51	55.66 ± 7.02	0.056
Cued	**15.5**	***rd1/wt***	**19.81 ± 4.04**	**61.20 ± 11.72**	**0.002**
Spatial	9.5	*wt/wt*	14.60 ± 1.99	35.73 ± 11.22	0.117
Spatial	9.5	*rd1/wt*	22.19 ± 3.77	27.97 ± 3.24	1.000
Spatial	15.5	*wt/wt*	28.04 ± 9.14	71.08 ± 10.97	0.117
Spatial	15.5	*rd1/wt*	29.51 ± 11.52	75.04 ± 16.54	0.396

Modeled escape latencies by age					
Phase	Genotype	*rd1* status	Age (9.5 m)	Age (15.5 m)	*p*
Cued	WT	*wt/wt*	17.53 ± 2.39	27.58 ± 5.51	0.651
Cued	WT	*rd1/wt*	18.18 ± 3.00	19.81 ± 4.04	1.000
Cued	5xFAD	*wt/wt*	54.20 ± 19.44	55.66 ± 7.02	1.000
Cued	5xFAD	*rd1/wt*	44.18 ± 6.77	61.20 ± 11.72	1.000
Spatial	WT	*wt/wt*	14.60 ± 1.99	28.04 ± 9.14	0.455
Spatial	WT	*rd1/wt*	22.19 ± 3.77	29.51 ± 11.52	1.000
Spatial	5xFAD	*wt/wt*	35.73 ± 11.22	71.08 ± 10.97	0.455
Spatial	**5xFAD**	***rd1/wt***	**27.97 ± 3.24**	**75.04 ± 16.54**	**0.002**

Modeled escape latencies by *rd1* status					
Phase	Genotype	Age (m)	*wt / wt* mice	*rd1 / wt* mice	*p*
Cued	WT	9.5	17.53 ± 2.39	18.18 ± 3.00	1.000
Cued	5xFAD	9.5	54.20 ± 19.44	44.18 ± 6.77	1.000
Cued	WT	15.5	27.58 ± 5.51	19.81 ± 4.04	1.000
Cued	5xFAD	15.5	55.66 ± 7.02	61.20 ± 11.72	1.000
Spatial	WT	9.5	14.60 ± 1.99	22.19 ± 3.77	0.455
Spatial	5xFAD	9.5	35.73 ± 11.22	27.97 ± 3.24	1.000
Spatial	WT	15.5	28.04 ± 9.14	29.51 ± 11.52	1.000
Spatial	5xFAD	15.5	71.08 ± 10.97	75.04 ± 16.54	1.000

Learning rates (slopes on model (log) link; s/day)				
Phase	Age	WT	5xFAD	*p*
Cued	**9.5 mo**	**− 0.315 ± 0.032**	**− 0.267 ± 0.032**	**0.024**
Cued	**15.5 mo**	**− 0.315 ± 0.032**	**− 0.267 ± 0.032**	**0.024**
Spatial	**9.5 mo**	**− 0.125 ± 0.015**	**− 0.077 ± 0.013**	**0.024**
Spatial	**15.5 mo**	**− 0.125 ± 0.015**	**− 0.077 ± 0.013**	**0.024**

Modeled escape latencies for each group with their corresponding *p* values (adjusted by Benjimini-Yakuteli false discovery correction). Data is presented as mean values ± standard error. Relevant effect sizes with *p* < 0.05 are discussed in the results section and shown in bold text here

### Search strategy selection in aged 5xFAD and WT mice

**Fig. 3:**
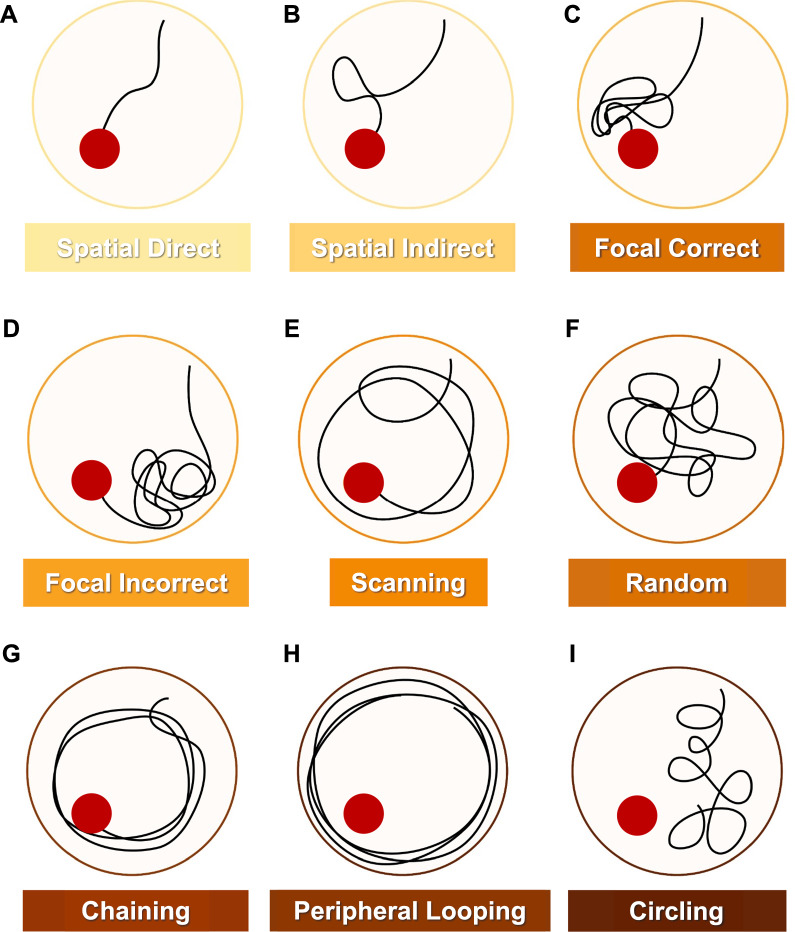
Search Strategy Assessment in MWM Testing. Search strategies were assigned to trials as described herein. **A** Spatial direct strategies imply that mice found the platform directly. **B** Spatial indirect strategies imply that mice find the platform within one loop. **C** Focal correct strategies involve a focused search adjacent to the platform. **D** An incorrect focal strategy implies a focused search in a small portion of the pool that does not contain the platform. **E** Scanning implies a platform search across the pool. **F** Random strategies imply a non-focused search in the entire pool. **G** Chaining involves a repetitive platform search inside the thigmotaxis zone, i.e., 15 cm from the rim of the pool. **H** Peripheral looping involves a platform search within the thigmotaxis zone. **I** Circling strategies involve movements in tight circles with directionality

**Fig. 4:**
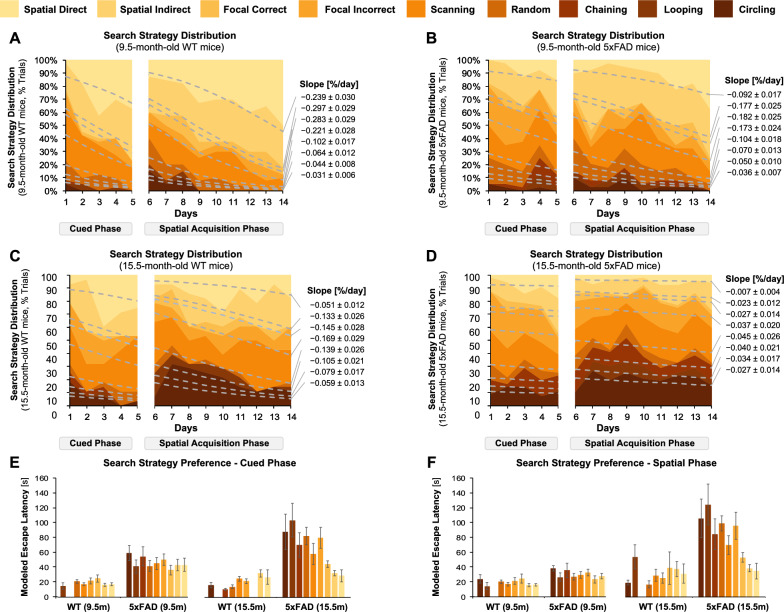
Search Strategy Selection of 5xFAD and WT mice. Search strategy preferences for **A** 9.5-month-old WT mice, B 9.5-month-old 5xFAD mice, **C** 15.5-month-old WT mice, and **D** 15.5-month-old 5xFAD mice. Data is presented as cumulative trials (% frequencies). Color legend for each strategy is presented at the top. Slopes for inter-strategy interfaces are indicated as mean ± standard errors of the mean (SEM) adjacent to each plot. For any given strategy, inter-strategy slopes were highest for 9.5-month-old WT mice, followed by 9.5-month-old 5xFAD mice, 15.5-month-old WT mice, and 15.5-month-old 5xFAD mice. **E** Histograms represent modeled escape latencies across search strategies mice adopt in the cued learning phase. **F** Histograms represent modeled escape latencies across mouse search strategies in the spatial acquisition phase. All strategies have similar escape latencies in 9.5-month-old mice. In contrast, shorter escape latencies were associated with structured strategies in 15.5-month-old mice. *Rd1* mutation did not have an impact on search strategy preferences

Next, we examined search strategies used by the mice to find the platform (Fig. 3). Brody and Holtzman have previously defined search strategies as 9 different spatial approaches that mice use to find a platform in MWM testing [31]. These strategies can be broadly classified as spatial strategies (most structured), non-spatial systemic strategies (intermediate), and repetitive strategies (least efficient). Search strategy analysis showed that 9.5-month-old WT mice used spatial strategies (40% trials on day 1, 80% trials on day 14) more frequently than non-spatial strategies (40% trials on day 1, 20% trials on day 14) or repetitive strategies (20% trials on day 1, 0% trials on day 14; Fig. 4A). In contrast, 9.5-month-old 5xFAD mice used non-spatial strategies (75% trials on day 1, 30% trials on day 14) more frequently compared to spatial (30% trials on day, 20% trials on day 14) to find the platform (Fig. 4B); repetitive strategies were used the least (< 5% trials on day 1 and day 14). These preferences indicate that 9.5-month-old 5xFAD mice use spatial strategies less frequently than age-matched WT mice. With aging, we noticed that WT mice preferred spatial, non-spatial, and repetitive strategies equally to reach the platform (33% trials on days 1 and 14, Fig. 4C). In contrast, 15.5-month-old 5xFAD mice used non-spatial strategies (50% trials on days 1 and 14) or repetitive strategies (30% trials on days 1 and 14) over spatial strategies (20% on days 1 and 14) to reach the platform (Fig. 4D). This shift suggests that aged mice prefer non-spatial and repetitive strategies and highlights poor learning outcomes in these mice. Estimated marginal means further revealed a transition from least to most structured strategies across all groups; this transition was less apparent for 5xFAD mice than age-matched WT mice and less apparent for older mice than younger mice.

We also determined the learning rates by calculating the interfaces' slopes between adjacent strategies. We found that all slopes were negative, confirming the preference for efficient strategies over time across all groups. Slopes for 9.5-month-old mice were steeper than slopes for 15.5-month-old mice (Fig. 4). These slopes indicate that younger mice adopt more structured strategies over time than their aged counterparts. 15.5-month-old 5xFAD mice showed little changes in strategy over time that corresponded to ≤ 1% transition per day. We further determined the effect of various factors on search strategy preferences during MWM testing. Estimated marginal means showed that preference for efficient strategies improved with successive testing days (*p* < 0.001; Table 4). Mice preferred efficient strategies more often in the cued phase but not in the spatial phase (*p* < 0.001; Table 4). We also found that age, genotype, test phase, and search strategy efficiency were associated with less efficient strategies during MWM testing (Table 4). We then examined if structured strategy preferences were associated with shorter escape latencies. Shorter escape latencies were not associated with search strategies in 9.5-month-old 5xFAD and WT mice (Fig. 4E, F). In contrast, shorter escape latencies were associated with more efficient search strategies in 15.5-month-old WT and 15.5-month-old 5xFAD mice.

**Table 4:** Effect Size Estimates for Search Strategy Preferences in MWM test

Effect	Estimate^a^	χ^2^ (df)	*p*
Age	0.075 ± 0.061	0.000 (1)	1.000
Genotype (5xFAD)	0.297 ± 0.065	0.000 (1)	1.000
Test Day	− 0.744 ± 0.070	193.095 (1)	< 0.001
Test Phase (Cued)	− 0.728 ± 0.087	150.088 (1)	< 0.001
Age × Genotype	0.217 ± 0.060	9.109 (1)	0.003
Age × Test Phase	− 0.098 ± 0.032	20.594 (1)	< 0.001
Genotype × Test Day	0.103 ± 0.040	7.654 (1)	0.006
Genotype × Test Phase	0.136 ± 0.050	12.304 (1)	< 0.001
Test Phase × Test Day	− 0.362 ± 0.069	47.111 (1)	< 0.001
Age × Genotype × Test Phase	− 0.054 ± 0.032	6.140 (1)	0.013
Age × Genotype × Strategy Efficiency	0.036 ± 0.103	59.461 (8)	< 0.001

Effect size estimates for search strategy preferences with corresponding *p* values, chi-squared values, and degrees of freedom. Positive values indicated that the effect was associated with higher structured strategies

^a^Calculated as the log of odds ratio ± standard error

**Fig. 5:**
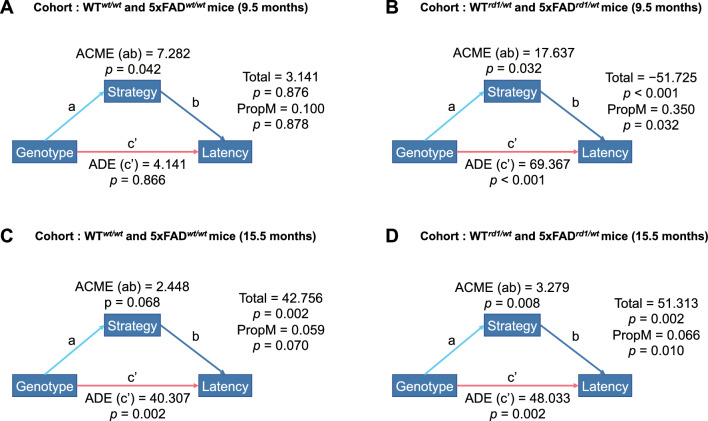
Moderated Mediation Analysis. **A** In 9.5-month-old 5xFAD^*wt/wt*^ and WT^*wt/wt*^ mice, strategy preferences contributed more to escape latencies than the genotype of the mouse model, but the effects of strategy and genotype were not significant. **B** In 9.5-month-old 5xFAD^*rd1/wt*^ and WT^*rd1/wt*^ mice, genotype contributed more to escape latencies than strategy preferences. **C** In 15.5-month-old 5xFAD^*wt/wt*^ and WT^*wt/wt*^ mice, genotype influenced escape latencies, but strategy preference did not. **D** In 15.5-month-old 5xFAD^*rd1/wt*^ and WT^*rd1/wt*^ mice, both genotype and strategy preference influenced escape latencies

Moderated mediation analysis further showed that search strategy preferences differently impacted escape latencies in mice based on age and *rd1* allele status. In 9.5-month-old WT^*wt/wt*^ and 5xFAD^*wt/wt*^ mice, efficient strategies contributed to lower escape latencies with an average causal mediation effect (ACME) of 7.28 (*p* = 0.042; Fig. 5A). Genotype, on the other hand, did not significantly contribute to latencies at this age with an average direct effect (ADE) of 4.14 (*p* = 0.866; Fig. 5A). The total effect of genotype on escape latencies was 3.14 (*p* = 0.876) and the effect of genotype mediated by strategy preference i.e., proportion of mediation (PropM) was 0.10 (*p* = 0.878). These values indicate that (a) 5xFAD mice and WT mice in this group showed similar latencies, and (b) strategy selection did not impact latencies in these mice. In 9.5-month-old WT^*rd1/wt*^ and 5xFAD^*rd1/wt*^ mice, genotype exerted a greater effect on escape latencies (ADE = 69.37 (*p* < 0.001); total effect = − 51.73 (*p* < 0.001)) than strategy selection (ACME = 17.64 (*p* = 0.032); PropM = 0.35 (*p* = 0.032) and indicated that (a) 9.5-month-old 5xFAD^*rd1/wt*^ mice showed higher escape latencies than age-matched WT^*rd1/wt*^ mice, and (b) search strategy selection contributes to higher escape latencies when *rd1* allele is present. In 15.5-month-old WT^*wt/wt*^ and 5xFAD^*wt//wt*^ mice, genotype continued to exert a greater effect on escape latencies (ADE = 40.31 (*p* = 0.002); total effect = 42.76 (*p* = 0.002)) but this was not the case for strategy (ACME = 2.45 (*p* = 0.068); PropM = 0.06 (*p* = 0.07)). These values indicate that older 5xFAD mice without the *rd1* allele performed worse in the MWM test irrespective of the selected strategy. In 15.5-month-old WT^*rd1/wt*^ and 5xFAD^*rd1/wt*^ mice, genotype significantly contributed to higher latencies with a greater effect size (ADE = 48.03 (*p* = 0.002); total effect = 51.31 (*p* = 0.002)) than strategy selection (ACME = 3.28 (*p* = 0.008); PropM = 0.07 (*p* = 0.01)).

Taken together, these findings show that (a) 5xFAD mice perform worse with age in the MWM test, (b) the presence of the *rd1* allele causes mice to perform worse in the MWM test at both ages due to inefficient strategy selection, and (c) older 5xFAD mice display a slower transition from least to most efficient strategies in the test. We presume these findings are due to other factors such as fatigue, lack of motivation, and slow swim speed, that should be explored in future studies.

### Progression of reference memory impairment in aged 5xFAD and WT mice

**Fig. 6:**
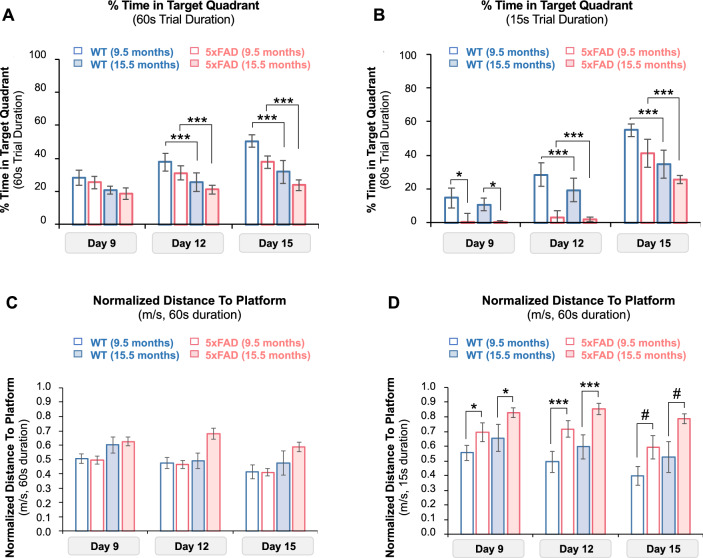
Probe Trial Metrics in MWM test. **A** Percent time in the target quadrant (Q) for 9.5-month-old 5xFAD mice (red hollow columns), 9.5-month-old WT mice (blue hollow columns), 15.5-month-old 5xFAD mice (red filled columns), and 15.5-month-old WT mice (blue filled columns) during the probe trial duration (60 s). Time in the target quadrant decreased with age. No differences in Q values were noted between 5xFAD and age-matched WT mice. **B** Q values during the first 15 s of the probe trial. Q values in the first 15 s of the trial showed higher resolution when compared with Q values at the end of the trial (60 s). *p* values estimated on model log scales. *p = 0.05, ^#^*p* = 0.029, ***p = 0.003. **C** Percentage of trials with zero time in target quadrant for all groups during the probe trial duration (60 s). **D** Percentage of trials with zero time in target quadrant for all groups in the first 15 s of the trial. **E** Normalized distance to platform (P, m/s) during probe trials. P values were similar for all groups in all probe trials. **F** Normalized distance to platform during the first 15 s probe trial. P values for 5xFAD mice were higher than P values for age-matched WT mice, but no differences were noted between age-matched 5xFAD and WT mice. *p* values estimated on model log scales. *p = 0.039, **p = 0.003, ***p < 0.001, ^#^*p* = 0.001

We evaluated 5xFAD and age-matched WT mice for reference memory impairment via probe trials (Fig. 6). We performed probe trials on days 9, 12, and 15 to confirm that mice could recall the platform location. For each probe trial, we introduced mice into the pool without the platform and allowed them to swim for the entire trial duration (60 s, Fig. 1A). Since mice were trained to locate the platform in the SW quadrant (days 6–8), we expected that time in the SW (target) quadrant would reflect the level of training in these mice. To assess reference memory, we compared three probe trial outcomes in 5xFAD and WT mice—percent time in the target quadrant (Q, Fig. 6A, B), percentage of trials with zero time in the target quadrant (Fig. 6C, D) and normalized distance (proximity) to the platform (P, Fig. 6E, F).

We first analyzed the percentage time in the target quadrant (Q_,_ Fig. 6A, B). According to Maei et al., Q is the most reported probe trial outcome [36]. Q values were significantly lower for 15.5-month-old mice than 9.5-month-old mice of the same genotype on days 12 and 15 (*p* < 0.003) and indicate that search accuracy in probe trials reduces with age (Fig. 6A). Pairwise testing also revealed significantly lower Q values for 5xFAD mice than age-matched WT mice on days 9 (*p* = 0.05) and 12 (*p* = 0.029). Post hoc estimation of marginal means showed that Q values increased with the duration of the probe trial and each successive test day but decreased with age in genotype-matched mice (Table 5). We also noted that Q values were lower for 5xFAD mice than age-matched WT mice with each successive test day. (Table 5). We then compared Q values in the first 15 s of each trial since mice tend to perform a more focused search in this timeframe [38]. Q values at 15 s were significantly lower for 15.5-month-old mice than 9.5-month-old mice of the same genotype on days 12 and 15 of the probe trials (*p* < 0.003; Fig. 6B). *Rd1* allele status did not impact Q values in this study. These values indicate that 5xFAD mice spend less time in the target quadrant than age-matched WT mice, and this duration further decreases with age.

**Table 5:** Effect Size Estimates for Percent Time Spent in Target Quadrant in Probe Trials

Effect	Estimate^a^	χ^2^ (df)	*p*	*R*^*2*^
Genotype (5xFAD)	**− 0.377 ± 0.077**	**23.974 (1)**	** < 0.001**	**0.253**
Age	**− 0.285 ± 0.076**	**14.059 (1)**	** < 0.001**	**0.109**
Test Duration	**0.315 ± 0.059**	**28.289 (1)**	** < 0.001**	**0.087**
Test Day	**0.260 ± 0.049**	**28.362 (1)**	** < 0.001**	** < 0.001**
Genotype × Test Duration	0.100 ± 0.054	20.946 (1)	< 0.001	0.051
Age × Test Duration	0.256 ± 0.056	3.447 (1)	0.063	0.011
Genotype × Test Day	**− 0.097 ± 0.048**	**4.020 (1)**	**0.045**	** < 0.001**

Effect size estimates for percent time spent in target quadrant with corresponding *p* values, chi-squared values, *R*^*2*^ values, and degrees of freedom

^a^Calculated as log odds ratio ± standard error

^b^Calculated on a log link ± standard error. Relevant effect sizes with *p* < 0.05 are discussed in the results section and shown in bold text here

In addition to Q values, we examined normalized distance to platform or proximity (P) as an additional outcome across probe trials. Compared to Q values, normalized distance to the platform is a more sensitive outcome in detecting inter-group differences, as Maei et al. retrospectively observed across 1600 probe trials [36]. Normalized distance to the platform is a continuous outcome with non-zero values since mice begin trials at a finite (non-zero) distance from the platform. Over the 60 s trial duration, all groups traveled similar distances from the platform across probe trials (Fig. 6C). In the first 15 s of the trial, 5xFAD mice traveled 1.5-fold greater distances than age-matched WT mice (Fig. 6D). Post hoc estimation of marginal means revealed that 15.5-month-old mice traveled longer distances when compared with 9.5-month-old mice of the same genotype (*p* = 0.012, Table 6). 5xFAD mice also traveled longer distances when compared with age-matched WT mice (*p* = 0.034) on all three probe trial days (Fig. 6D). Genotype, test day, and test duration collectively impacted normalized distance to platform in probe trials (Table 6). *Rd1* allele status did not impact P values in this study.

**Table 6:** Effect Size Estimates for Normalized Distance to Platform in Probe Trials

Effect	Estimate^a^	χ^2^ (df)	*p*
Genotype (5xFAD)	**− 0.115 ± 0.033**	**10.854 (1)**	** < 0.001**
Test duration	**0.106 ± 0.033**	**58.906 (1)**	** < 0.001**
Age	**− 0.061 ± 0.013**	**10.410 (1)**	**0.001**
Test day	**− 0.103 ± 0.013**	**21.351 (1)**	** < 0.001**
Genotype × Test day	0.111 ± 0.034	4.468 (1)	0.035
Genotype × Test duration	0.028 ± 0.013	15.349 (1)	< 0.001

Estimates are reported with corresponding *p* values, chi-squared values, and degrees of freedom

^a^Calculated as log odds ratio ± standard error

^b^Calculated on a log link ± standard error. Relevant effect sizes with *p* < 0.05 are discussed in the results section and shown in bold text here

In conclusion, probe trial outcomes show a deteriorating performance with age and that 5xFAD mice explored the target quadrant less frequently and traveled longer distances to find the platform than age-matched WT mice. We also noted enhanced trial resolution in the first 15 s when compared with the entire trial duration.

### Changes in plasma and brain Aβ levels with age in 5xFAD mice

**Fig. 7:**
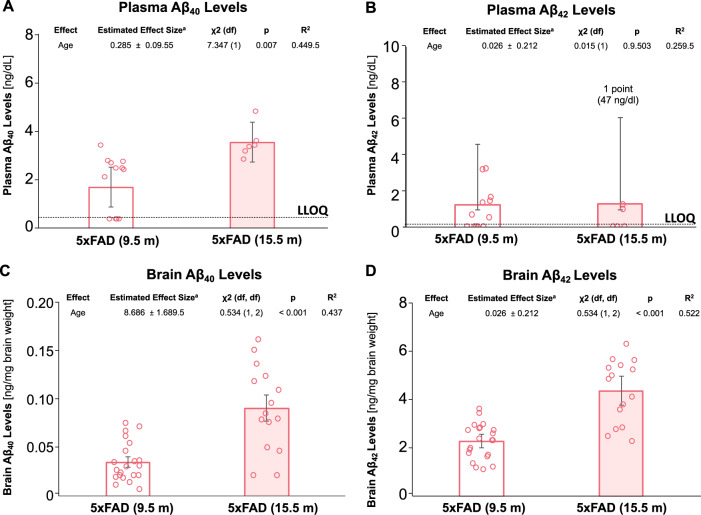
Plasma and Brain Aβ levels in 5xFAD Mice. **A** Median plasma Aβ_40_ levels (ng/dL) were significantly higher for 15.5-month-old 5xFAD mice (red filled column) than 9.5-month-old 5xFAD mice (red hollow column). **B** Median plasma Aβ_42_ levels (ng/dL) were similar for 9.5-month-old 5xFAD mice (red hollow column) and 15.5-month-old 5xFAD mice (red filled column). Error bars in A and B show the interquartile range. Dashed lines indicated lower limits of quantitation. **C** Mean brain Aβ_40_ levels (ng/mg brain weight) were significantly higher for 16-month-old 5xFAD mice (red filled column) than 9.5-month-old 5xFAD mice (red hollow column). **D** Mean brain Aβ_42_ levels (ng/mg brain weight) in 9.5-month-old 5xFAD mice (red hollow column) and 15.5-month-old 5xFAD mice (red filled column). No significant differences were noted between the two groups. Red hollow circles represent individual data points. The dotted lines in A and B show the lower limit of quantitation (LLOQ) for Aβ. Error bars in A and B show the interquartile range, and errors in C and D show the standard error of the mean (SEM)

We then determined Aβ_40_ and Aβ_42_ levels in brain tissue and plasma samples of 9.5-month-old and 15.5-month-old 5xFAD mice by ELISA (Fig. 7). We did not test samples from WT mice since they do not express human Aβ protein. Median plasma Aβ_40_ levels were twofold higher (*p* = 0.007) in 15.5-month-old 5xFAD mice (3.6 ng/dL) compared to levels from 9.5-month-old 5xFAD mice (1.75 ng/dL; Fig. 7A). In contrast, median plasma Aβ_42_ levels were not significantly different between 9.5-month-old (1.3 ng/dL) and 15.5-month-old 5xFAD mice (1.4 ng/dL; Fig. 7B). Mean brain Aβ_40_ levels increased 2.5-fold (*p* < 0.001): from 36.3 ± 5.2 ng/mg brain tissue in 9.5-month-old 5xFAD mice to 92.7 ± 14.0 ng/mg brain tissue in 15.5-month-old 5xFAD mice (*p* < 0.001; Fig. 7C). Mean brain Aβ_42_ levels also increased by twofold (*p* < 0.001): from 2.3 ± 0.3 ng/mg brain tissue in 9.5-month-old 5xFAD mice) to 4.4 ± 0.6 ng/mg brain tissue in 15.5-month-old 5xFAD mice (Fig. 7D). Taken together, we observed an age-dependent increase in Aβ levels in multiple compartments of 5xFAD mice.

### Barrier integrity in aged 5xFAD and WT mice

**Fig. 8:**
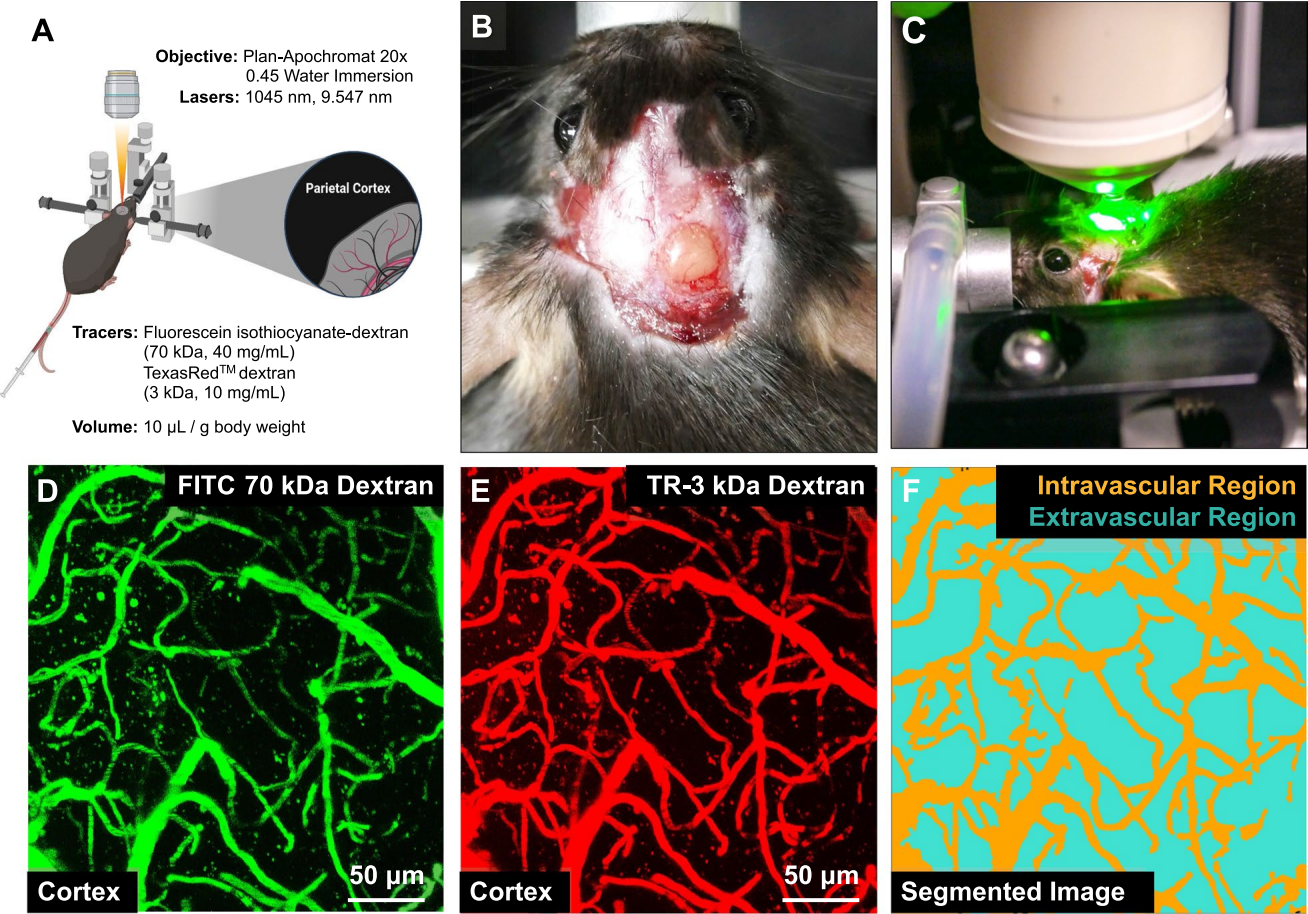
In vivo Two-Photon Microscopy Imaging of 5xFAD and WT mice. **A** Schematic graphic showing an overview of the in vivo imaging setup. **B** Image showing a mouse head with an acutely installed cranial window. **C** Image of an anesthetized mouse secured on the microscopy board for in vivo microscopy. **D** MIP image of cortical vessels infused with FITC-70 kDa dextran (green). **E** MIP of cortical vessels infused with TR-3 kDa dextran (red). **F** Segmented image obtained from the Zen Intellesis module that shows intravascular (orange) and extravascular (cyan) regions

**Fig. 9:**
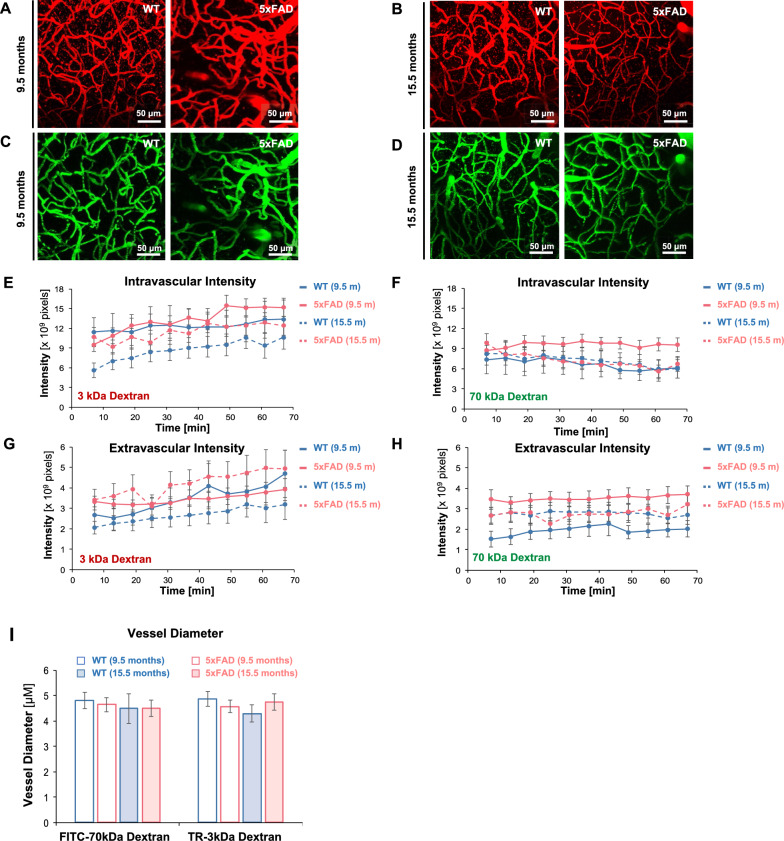
Distribution of fluorescent-labeled dextrans in 5xFAD and WT Mice. **A**–**D** Representative MIP images from age-matched 5xFAD and WT mice for each dextran. **E** Intravascular sum intensity of FITC-70 kDa dextran MIP images over 70 min of in vivo imaging. **F** Extravascular sum intensity for FITC-70 kDa dextran MIP images. **G** Intravascular sum intensity of 3 kDa dextran MIP images. **H** Extravascular sum intensity of 3 kDa dextran. **I** Vessel diameters were measured from in vivo MIP images with FITC-70 kDa dextran and TR-3 kDa dextran labeled vessels. Legend: 9.5-month-old WT mice (blue hollow column), 9.5-month-old 5xFAD mice (red hollow column), 15.5-month-old WT mice (blue filled column), and 15.5-month-old 5xFAD mice (red filled column). Sample sizes for TR-3 kDa dextran-labeled vessels: 9.5-month-old WT mice (*n* = 9), 9.5-month-old 5xFAD mice (*n* = 11), 15.5-month-old WT mice (*n* = 12), 15.5-month-old 5xFAD mice (*n* = 11). Sample sizes for FITC-70 kDa dextran-labeled vessels: 9.5-month-old WT mice (*n* = 8), 9.5-month-old 5xFAD mice (*n* = 11), 15.5-month-old WT mice (*n* = 11), 15.5-month-old 5xFAD mice (*n* = 11). Error bars indicate standard errors of the mean (SEM) for each measurement

We determined the in vivo distribution of fluorescent-labeled dextrans in 5xFAD and WT mice using two-photon microscopy. In brief, we installed cranial windows and injected a combination of FITC-70 kDa dextran and TR-3 kDa dextran into the tail vein of anesthetized 5xFAD mice and WT mice (Fig. 8). We acquired sequential Z-stacks (150 μm-thick) of cerebrovascular networks every 7 min for 70 min and generated maximum intensity projection (MIP) images for each Z-stack. Figure 9A–D show representative MIP images of fluorescent-labeled dextrans in the brain vasculature of WT or 5xFAD mice. We segmented MIP images into intra- and extravascular compartments and used sum intensities to assess dextran distribution in these compartments as a measure of barrier integrity. Post hoc assessment of in vivo data shows that age, compartment type, tracer size, genotype, and time exerted minor (effect size estimate < 0.1) but significant (*p* < 0.05) effects on dextran intensities (Table 7). Figure 9E–H show dextran intensities within the intra- and extravascular compartments across all groups. Intravascular TR-3 kDa dextran intensities increased with time but did not vary with age and genotype (Fig. 9E). Intravascular FITC-70 kDa dextran intensities did not change with age, genotype, and time (Fig. 9F). Extravascular TR-3 kDa dextran intensities increased with time but did not change with age or genotype (Fig. 9G). Extravascular FITC-70 kDa dextran intensities did not change with age, genotype, and time in these mice (Fig. 9H). We further measured capillary diameters for each dextran but found no significant differences across groups (Fig. 9I). Post hoc analysis of estimated marginal means showed that age and tracer type combined affected vessel diameters (*p* = 0.043; Table 8). Pairwise comparison did not show significant differences between vessel diameters across groups for a specific tracer. Taken together, our in vivo image analysis shows that extra- and intravascular intensities for both FITC-70 kDa and TR-3 kDa, as well as vessel diameters, did not significantly change with time, age, or genotype for both tracers.

**Table 7:** Effect Size Estimates for In vivo Fluorescence Intensities

Effect	Estimate^a^	χ^2^ (df)	*p*
Age	− 0.696 ± 0.481	2.093 (1)	0.148
Age × Extravascular Compartment	0.052 ± 0.011	24.216 (1)	< 0.001
Age × Extravascular Compartment × Genotype	− 0.009 ± 0.011	0.718 (1)	0.397
Age × Extravascular Compartment × Genotype × Time	0.031 ± 0.010	9.143 (1)	0.002
Age × Genotype	0.102 ± 0.480	0.045 (1)	0.832
Age × Genotype × Time	− 0.024 ± 0.011	5.294 (1)	0.021
Age × Genotype × Tracer Size	− 0.066 ± 0.012	31.295 (1)	< 0.001
Age × Time	− 0.011 ± 0.011	1.026 (1)	0.311
Age × Tracer Size	0.018 ± 0.014	1.688 (1)	0.194
Age × Tracer Size × Time	− 0.024 ± 0.010	5.575 (1)	0.018
Extravascular Compartment	− 0.522 ± 0.011	2446.141 (1)	< 0.001
Extravascular Compartment × Genotype	− 0.033 ± 0.011	9.762 (1)	0.002
Extravascular Compartment × Genotype × Time	− 0.002 ± 0.010	0.028 (1)	0.867
Extravascular Compartment × Time	0.018 ± 0.010	3.186 (1)	0.074
Extravascular Compartment × Time	0.011 ± 0.010	1.212 (1)	0.271
Extravascular Compartment × Tracer Size × Time	0.025 ± 0.010	6.222 (1)	0.013
Extravascular Compartment × Tracer Size	0.063 ± 0.010	38.594 (1)	< 0.001
Genotype	0.325 ± 0.480	0.458 (1)	0.499
Genotype × Time	− 0.001 ± 0.011	0.018 (1)	0.894
Genotype × Tracer Size	0.024 ± 0.013	3.369 (1)	0.066
Time	0.023 ± 0.011	4.831 (1)	0.028
Tracer Size	− 0.157 ± 0.013	138.653 (1)	< 0.001
Tracer Size × Time	− 0.057 ± 0.010	31.735 (1)	< 0.001

Estimates are reported with corresponding *p* values, chi-squared values, and degrees of freedom

^a^Calculated on a log link. Data is presented as mean values ± standard error

**Table 8:** Effect Size Estimates for Vessel Diameters

Effect	Estimate	χ^2^ (df)	p
Age	− 0.027 ± 0.021	1.605 (1)	0.205
Tracer Size	− 0.012 ± 0.006	3.836 (1)	0.050
Genotype (5xFAD)	0.015 ± 0.021	0.472 (1)	0.492
Age × Tracer Size	− **0.013 ± 0.006**	**4.115 (1)**	**0.043**

Estimates are reported with corresponding *p* values, chi-squared values, and degrees of freedom. Data is presented as mean ± standard error. Relevant effect sizes with *p* < 0.05 are discussed in the results section and shown in bold text here

## Discussion

Preclinical models are a first step in understanding mechanisms underlying central nervous system disorders such as AD and offer researchers the potential to discover and test new approaches and screen novel therapeutics with the goal of successful translation into the clinic [42, 43]. However, mouse AD models develop AD pathology to varying degrees with age, which often makes it challenging to obtain relevant preclinical findings on safety and efficacy [6]. Therefore, it is critical to understand how AD pathology changes with age, which will help inform the appropriate age range for preclinical treatment and efficacy studies. In the present study, we characterized 5xFAD and age-matched WT mice at 9.5 and 15.5 months of age with respect to cognition, Aβ levels, and blood–brain barrier integrity.

Ages chosen for this study are equivalent to middle-aged (38–47 years) [44] and older (> 55 years) familial AD/early-onset AD patients. Although 5xFAD mice are prone to higher mortality rates, our experience of housing 300 + 5xFAD mice and reports from other research groups indicate that 5xFAD mice tend to live up to 15 months of age and longer [17, 45]. Like other AD models, some of the age-related changes in 5xFAD mice do not start until the age of 12 months, which justifies the use of 15-to-16-month-old mice for our study [46–48].

We used male 5xFAD mice in the study. Although female 5xFAD mice exhibit worse Aβ pathology than males due to estrogen-regulated hAPP overexpression, sex differences in the vessel architecture have been previously noted for older 5xFAD mice [49, 50]. Recent vessel painting studies show that male 5xFAD mice show reduced vessel density associated with the middle cerebral artery regions and increased leakage hotspots from 4 to 12 months of age, but this was not observed in the case of female 5xFAD mice [50]. Studies by Zukhov et al. and Giannoni et al. show varying results on barrier integrity in female 5xFAD mice below and above 9 months of age, indicating that the effect of menopause cannot be completely excluded [21, 51]. In this regard, the use of male 5xFAD mice excludes the effect of the estrous cycle in mice between 9 and 16 months of age. We found that 9.5- and 15.5-month-old 5xFAD mice display significant spatial and reference memory deficits compared to age-matched WT mice. We also observed an age-dependent increase in cognitive deficits and a reduced preference for structured search strategies in the MWM assay in 5xFAD mice. As expected, we found that 15.5-month-old 5xFAD mice have higher Aβ_40_ and Aβ_42_ levels in the brain compared to 9.5-month-old 5xFAD mice. We also found that plasma Aβ_40_ levels also increased with age in 5xFAD mice. Further, we demonstrate intra- and extravascular changes in the in vivo distribution of dextrans, and vessel diameters are not significantly different between aged 5xFAD and WT mice.

### Cognitive deficits

We found that, with age, 5xFAD mice exhibit progressing spatial memory deficits compared to age-matched WT mice. The use of statistical models allowed us to draw relevant inferences from experiments. These deficits are reflected as a gap between mean escape latencies for 9.5-month-old 5xFAD and WT mice. This gap widens as mice reach 15.5 months of age. A similar age-dependent increase in spatial memory deficits has been reported for 5xFAD and WT mice at 9 and 15 months of age [52]. Factors such as albinism, motor impairments, muscle tone, and retinal degeneration partly contribute to increased latencies and higher failure rates in older 5xFAD and WT mice [14, 53]. We noted that the gap between modeled escape latencies for 5xFAD and age-matched WT mice is larger than the gap between mean escape latencies for the two groups. This is to be expected, since analysis of right-censored data ignoring the truncation at 60 s introduces a confounding artifact and calls into question all analyses that do not consider the unavoidable fact that stopping a test at 60 s is not the same result as a 60 s latency and should not be treated as such. In this regard, mean and modeled escape latency data presented in the study confirm that 15.5-month-old 5xFAD mice take significantly longer to find the platform in the MWM test than age-matched WT mice, indicating cognitive impairment. The presence of the *rd1* allele appeared to exert effects on MWM performance and the relationship between search strategy and performance. However, our sample turned out to be unbalanced for allele distribution, and some combinations had a very small number (n = 2 or 3). Thus, we would not infer beyond stating that this allele and other alleles that affect vision should be taken into consideration if using behavioral testing that relies on visual cues, such as MWM. In our study, given animal numbers, we can conclude that *rd1* may be a confounding variable, and further work would be more conclusive using *rd1*-free animals. Our work does indicate that interaction between what is deemed primarily “cognitive” traits, e.g., AD transgenes, and “non-cognitive” traits, e.g., visual acuity, merits further investigation.

We also found that 5xFAD mice used non-structured search strategies more frequently compared to their age-matched WT mice. These findings agree with previous reports of 3-to-12-month-old 5xFAD mice using non-spatial/repetitive strategies more frequently than WT mice at the same age [48, 52]. Interestingly, both groups of 15.5-month-old mice, WT and 5xFAD, adopted more structured search strategies to find the platform than their 9.5-month-old counterparts. Moreover, shorter escape latencies were associated with more structured strategies in 15.5-month-old WT mice but with less structured strategies in 5xFAD mice at the same age. Probe trial outcomes further confirmed that 15.5-month-old 5xFAD mice explored the target quadrant less frequently than age-matched WT mice. These outcomes suggest that 15.5-month-old 5xFAD mice engage less with MWM cues compared to age-matched WT mice and younger (9.5-month-old) 5xFAD mice. Further, mice search the platform more intensely in the first 15 s of a probe trial where the platform is removed. After about 15 s, when they cannot locate the platform, mice expand their search to the rest of the pool. Thus, analyzing the first 15 s accounts for a more sensitive measurement of inter-group variability during probe trials. Similar findings have been reported elsewhere [36]. Mice may "lose interest" in searching a specific area when the platform is removed and broaden their search over time. This underscores the importance of selecting the appropriate interval for assessing probe trial performance, which may vary across different mouse models.

### Brain & plasma Aβ Levels

5xFAD mice develop Aβ pathology earlier than other transgenic strains, such as Tg2576 (~ 3 months), TgSwDI (3 months), TgCRND8 (5 months), 3xTg-AD (6 months), or APP (V717I) mice (10 months) [6]. As expected, we found that Aβ_40_ and Aβ_42_ brain levels increase with age in 5xFAD mice. We also found that plasma Aβ_40_ increases with age in 5xFAD mice, whereas Aβ_42_ levels remained unchanged. Apart from these findings, an age-dependent increase in plasma and brain Aβ levels in 4-to-8-month-old 5xFAD mice has been reported [17, 53]. We did not observe any significant associations between plasma and brain Aβ levels.

### Barrier Integrity

5xFAD mice have not been studied extensively for blood–brain barrier integrity [6]. The existing studies show various changes differing in the degree of barrier dysfunction. For example, Giannoni et al*.* reported microvascular leakage ‘hotspots’ for FITC-Albumin (66.3 kDa) in 5xFAD mice at 9 and 12 months of age [21]. In 5-to-7-month-old 5xFAD mice, blood flow stalls in a small fraction (1.5–2%) of brain capillaries [19]. Fibrinogen (340 kDa) deposits in less than 2% of the total cortical area in 3-month-old and 6-month-old 5xFAD mice [54, 55]. Electron microscopy indicates that 8-month-old 5xFAD mice have short and tortuous capillaries with shorter tight junctions (twofold reduction; p < 0.05) compared to tight junctions in long, tubular capillaries in 8-month-old WT mice [56, 57]. 9-month-old 5xFAD capillaries had a 50% reduced signal for GLUT-1 and ZO-1 immunofluorescence when compared to age-matched WT mice [58]. MRI showed occasional cerebral microbleeds in 15.5-month-old 5xFAD mice and age-matched WT mice [58]. Data from several studies indicate that changes at the blood–brain barrier in AD are subtle and complex with inconsistencies observed in blood–brain barrier dysfunction across AD animal models [6]. Data on barrier dysfunction in aged 5xFAD mice is lacking.

More recently, a lack of difference in blood–brain barrier integrity between 7-and-10-month-old 5xFAD and age-matched WT mice was reported [51]. Specifically, no differences existed in vesicular transport of fluorescently labeled albumin across pial vessels, penetrating arterioles, and capillaries in 5xFAD and age-matched WT mice. Both strains of mice had similar capillary diameters following whisker stimulation (neurovascular coupling) and similar levels of extravascular intensities after intravenous injections of sodium fluorescein (paracellular leakage). Our in vivo findings are consistent with these observations by using a different analytical approach. Zhukov et al. [51] assessed barrier leakage by (1) averaging parenchymal intensities for 4–5 ROIs, and (2) excluded ROIs that were next to pial vessels and Aβ plaques. Based on this approach, the authors concluded that the blood–brain barrier is preserved in aged female 5xFAD mice. In our study, we used the AI-based approach to determine fluorescence intensities in the entire Z-stack that could be deemed extravascular. This method provides a complete and unbiased assessment of fluorescence intensities and considers all heterogeneities. Using this method, we found that intra- and extravascular dextran fluorescence intensities remained unchanged, suggesting that the blood–brain barrier is still intact in 9.5- and 15.5-month-old 5xFAD mice compared to age-matched WT mice.

We further discuss several aspects and limitations of our current methodology and analysis. First, it is unknown why TR-3 kDa shows increased intravascular fluorescence intensities over time. This increase, however, is not significant. It is possible that TR-3 kDa dextran rapidly diffuses into perivascular and parenchymal spaces, and perivascular TR-3 kDa was miscategorized as intravascular in this analysis. If this were to be the case, one would expect larger diameters for TR-3 kDa labeled vessels compared to FITC-70 kDa labeled vessels, but we did not measure any significant differences between these two vessel diameters. We acknowledge that measurement of blood/brain/urine concentrations for each tracer would allow us to determine volumes of distribution, brain/serum ratio, and clearance values and draw insights into how intravascular/extravascular tracer intensities correlate with tracer concentrations in other tissues.

Second, what contributes to variations in FITC-70 kDa dextran and TR-3 kDa dextran distribution in the brain is unclear. Multiple previous pharmacokinetic and longitudinal imaging studies have noted size-based variations in dextran distribution in the brain. Higher fluorescence intensities for 40 kDa dextrans than 70 kDa dextrans over time in 9–11-week-old C57BL/6 mice have been reported [59]. Furthermore, other work reported the highest fluorescence intensities for sodium fluorescein (0.3 kDa) compared to 3 kDa FITC-dextran and 40 kDa FITC-dextran over time in young (5-month) vs old (24-month) C57BL/6 mice [60]. In general, dextran conjugates with a molecular weight below 15,000 Daltons are filtered unrestricted, and consequently, the elimination half-life of dextran 1 is relatively short (2 h) and that of dextran 40 (10 h) or dextran 60 (42 h) much longer [61]. The presence of different amounts of conjugated fluorophores (i.e., 0.3 mol TexasRed per mole 3 kDa dextran, 0.003–0.02 mol FITC per mole 70 Da dextran) also makes it impossible to do a like-to-like comparison for their clearance over time.

Third, our segmentation method does not entirely exclude all puncta from all non-training images. Puncta, if any, that were included in the intravascular compartment were likely due to their high intensities, which, in some instances, also represented segments of small vessels between adjacent red blood cells. We cannot completely exclude the possibility that some of these puncta likely represent tracers taken up by brain cells. Characterizing these puncta for their distribution (number, % area, mean intensity) would be an interesting endpoint to analyze and identify how it might complement fluorescence intensity datasets. Fourth, our segmentation approach is not sensitive enough to classify between intravascular, perivascular, and extravascular fluorescence. It is possible that fluorescence intensities in the perivascular region were miscategorized as intravascular fluorescence intensities in this analysis. Even though the barrier might be compromised, these limitations prevent us from resolving significant differences using the current method.

## Conclusion

In the present study, we characterized 5xFAD mice and age-matched WT at 9.5 and 15.5 months of age for cognition, Aβ levels, and blood–brain barrier integrity. The ages chosen for this study allow us to assess the impact of aging on Aβ accumulation using the 5xFAD mouse model. Further, our data show that aging does not enhance microvascular dysfunction in the 5xFAD mouse model, potentially due to abundant non-vascular (Aβ42) accumulation in these mice [18]. There are limited studies that provide such a perspective using aged 5xFAD mice, indicating that the choice of animal model can have a significant impact on the study outcome. Thus, our study fills an obvious gap in the literature of studies that characterize AD animal models for microvascular dysfunction. Changes in spatial memory deficits and Aβ levels in 5xFAD mice are age-dependent, and thus, age should be carefully considered when planning preclinical studies in 5xFAD mice. Using in vivo MP imaging, no significant differences in barrier integrity were observed in aged mice. However, lower molecular weight tracers, combined with advanced imaging techniques that visualize the entire brain, may be better suited to assess barrier integrity. In this regard, our study raises awareness of the limitations in current imaging and analytical methods that are widely used but are unable to detect subtle forms of barrier dysfunction. Lastly, our study informs researchers in the brain barriers field to carefully select their animal models and techniques to fit their studies. Overall, it remains to be determined if the blood–brain barrier is truly preserved in the 5xFAD mouse, a model with accelerated AD pathology.

## Data Availability

The raw and analyzed datasets generated for this study are available from the corresponding author on reasonable request.

## Abbreviations

ACME: Average causal mediated effect
AD: Alzheimer's disease
ADE: Average direct effect
AICc: Akaike Information Criterion (AIC) with a correction for small sample sizes
APP: Amyloid Precursor Protein
Aβ: Amyloid beta
CAA: Cerebral Amyloid Angiography
FITC: Fluorescein Isothicyanate
FITC-70 kDa dextran: Fluorescein-Isothiocyanate 70 kDa dextran
LLOQ: Lower Limit of Quantitation
MIP: Maximum Intensity Projection
MMRRC: Mutant Mouse Resource & Research Center
MRI: Magnetic Resonance Imaging
MWM: Morris Water Maze
NE: Northeast
P value: Proximity or normalized distance to platform
PET: Positron Emission Tomography
PropM: Proportion of mediation
Q value: Percentage time in target quadrant
rd1: Retinal degeneration 1
SW: Southwest
TR: TexasRed
TR-3 kDa: Texas RedTM 3 kDa dextran
WT: Wild-Type
WTrd1/wt: WT mice with heterozygous rd1 allele
WTwt/wt: WT mice without rd1 allele
5xFADrd1/wt: 5XFAD mice with heterozygous rd1 allele
5xFADwt/wt: 5XFAD mice without rd1 allele
